# Optimizing an mHealth Program to Promote Type 2 Diabetes Prevention in High-Risk Individuals: Cross-Sectional Questionnaire Study

**DOI:** 10.2196/45977

**Published:** 2023-10-16

**Authors:** Edgar Ross, Ebaa Al Ozairi, Naeema Al qabandi, Robert Jamison

**Affiliations:** 1 Atrius Healthcare Harvard Medical School Burlington, MA United States; 2 Dasman Diabetes Institute Kuwait City Kuwait; 3 Brigham and Women's Hospital Harvard Medical School Boston, MA United States

**Keywords:** SMS, short text message interventions: mHealth, smartphone, type 2 diabetes prevention, social media, friends and family, percolation theory, diabetes, prevention, risk, development, pilot study, social network, theory modeling, disease control, initiative

## Abstract

**Background:**

We evaluated the outcomes of a pilot SMS text messaging–based public health campaign that identified social networking nodes and variations of response rates to develop a list of variables that could be used to analyze and develop an outreach strategy that would maximize the impact of future public health campaigns planned for Kuwait. Computational analysis of connections has been used to analyze the spread of infectious diseases, dissemination of new thoughts and ideas, efficiency of logistics networks, and even public health care campaigns. Percolation theory network analysis provides a mathematical alternative to more established heuristic approaches that have been used to optimize network development. We report on a pilot study designed to identify and treat subjects at high risk of developing type 2 diabetes mellitus in Kuwait.

**Objective:**

The aim of this study was to identify ways to optimize efficient deployment of resources and improve response rates in a public health campaign by using variables identified in this secondary analysis of our previously published data (Alqabandi et al, 2020). This analysis identified key variables that could be used in a computational analysis to plan for future public health campaigns.

**Methods:**

SMS text message screening posts were sent inviting recipients to answer 6 questions to determine their risk of developing type 2 diabetes mellitus. If subjects agreed to participate, a link to the Centers for Disease Control and Prevention prediabetes screening test was automatically transmitted to their mobile devices. The phone numbers used in this campaign were recorded and compared to the responses received through SMS text messaging and social media forwarding.

**Results:**

A total of 180,000 SMS text messages through 5 different campaigns were sent to 6% of the adult population in Kuwait. A total of 260 individuals agreed to participate, of which 153 (58.8%) completed the screening. Remarkably, 367 additional surveys were received from individuals who were not invited by the original circulated SMS text messages. These individuals were invited through forwarded surveys from the original recipients after authentication with the study center. The original SMS text messages were found to successfully identify influencers in existing social networks to improve the efficacy of the public health campaign.

**Conclusions:**

SMS text messaging–based health care screening campaigns were found to have limited effectiveness alone; however, the increased reach through shared second-party forwarding suggests the potential of exponentially expanding the reach of the study and identifying a higher percentage of eligible candidates through the use of percolation theory. Future research should be directed toward designing SMS text messaging campaigns that support a combination of SMS text message invitations and social networks along with identification of influential nodes and key variables, which are likely unique to the environment and cultural background of the population, using percolation theory modeling and chatbots.

## Introduction

Countries belonging to the Gulf Cooperation Council (GCC), which include Saudi Arabia, Bahrain, Kuwait, Qatar, Oman, and the United Arab Emirates, have collectively experienced a rapid increase in prosperity since the discovery of oil [1]. This wealth has led to rapid lifestyle changes that include a trend toward more sedentary activities and increasing calorie consumption. This trend has contributed to a substantial increase in the incidence of noncommunicable diseases, including type 2 diabetes mellitus (T2DM). Obesity is a key risk factor for a number of noncommunicable diseases, with the principal one being insulin resistance and the development of T2DM. This trend led to 4 of the GCC countries (Kuwait, Saudi Arabia, Qatar, and Bahrain) being listed in the top 10 countries in 2012 with the highest rates of diabetes in the world. This trend is not just confined to the GCC. The association between improved standard of living and obesity can be found throughout the entire Middle East and in North African countries. Even lower income countries in the Middle East are exhibiting the same trends, and all of them will face problematic increases in the incidence of T2DM in the upcoming decades. The estimate of the prevalence of T2DM in the Middle Eastern and North African region after age adjustment is 10.9%, the highest of any region in the world [2]. Even low-income countries are not exempt from this trend, as three-quarters of all people with T2DM live in low- to middle-income countries, which will experience the greatest increase in the incidence of T2DM in the coming years as their economies grow [3]. Despite these statistics, organized attempts to prevent T2DM continue to lag. Globally, 45.8% of people with diabetes are undiagnosed, 83.8% of whom live in low- to middle-income countries [4].

The root cause of these trends is multifactorial. Beyond environmental factors, other contributing factors include unhealthy diet, inactive lifestyle, genetics, and disposable income often leading to dietary shifts toward more western preferences, including refined carbohydrates, low fiber, and high saturated fats. Lifestyle changes come from eating outside the home with preference toward western foods, a more sedentary life with the introduction of television and computer games, migrant workers being hired for labor-intensive occupations, and rapid urbanization, which emphasizes automobiles and the reduction of open spaces that support physical activity [5,6].

Social and cultural mores can also inhibit formal exercise programs by proscribing men and women from wearing suitable clothing for exercise and requiring that women be accompanied by related men in public. In addition, many low-income regions in the world have a hospitality culture where food consumption is the center of social activities, and obesity is considered to be a sign of growing financial affluence [2].

Despite these trends, the overwhelming consensus in evidence-based literature is that T2DM can be managed, reversed, and even prevented by the modification of lifestyle choices [7]. Many public health initiatives fail because they are unable to find and educate the general public on disease prevention, which includes behavioral changes that are needed to avoid otherwise inevitable outcomes. Traditional approaches have used public service announcements or have relied on primary care screening to identify patients at risk. These traditional approaches are either not well-developed or have substantial upfront costs, both of which are not viable options in low-income countries with limited funding [8]. Novel cost-effective public health initiatives are needed to address this slow developing epidemic. One approach that has been suggested is leveraging the ubiquitous smartphone to transmit preventative health messages in collaboration with key or established health care resources with supporting infrastructure [9,10].

A confirmed diagnosis of T2DM is preceded by a decade’s long latency period and even more elapsed time before the onset of complications from diabetes. This provides a substantial opportunity to intervene and potentially reverse the behaviors that have led to the alarming increase of diabetes in Kuwait and countries of similar economic status [11]. In 2017, Kuwait had a population of 3,024,558, of which 446,635 were diagnosed with diabetes and an additional 75,000 were reportedly undiagnosed. The long latency period suggests that those individuals most at risk are also the ones most likely to use social media regularly.

Globally, smartphone penetration has exceeded 80% and is the leading way to access the internet throughout the world. This is especially true for low-income countries where hardwired internet access infrastructure does not exist. Leveraging this resource could be a very cost-effective way to reach individuals at risk for T2DM if used efficiently. The phone survey approach has already been implemented using different platforms, including interactive voice response, telephone surveys, computer-aided automated surveys, and SMS text messaging approaches, to survey public health. SMS text messaging has very little data supporting its use in this context [9]. Previous studies have reviewed the low response rates and waning interest in follow-through for mobile health (mHealth) initiatives [12,13]. Management costs of a campaign, seasonal population shifts because of climate conditions, and religious holidays can be success-limiting barriers. We used the Centers for Disease Control and Prevention (CDC) type 2 risk assessment as the screening tool to develop a smartphone app called StopType2 [14]. It has been translated into Arabic and was distributed through an automated SMS text message using a national cell phone database.

Although established social media groups as a sole platform are compelling because of their widespread popularity, their use alone could be ineffective because of the distraction of many different and conflicting posts and advertisements, lack of trust, costs to public health agencies, and difficulty recruiting key influencers. Percolation theory, which was initially derived from the theory of the properties of classical particles interacting with a random medium, has been used to explain and predict the future interactions of networks clustered in random components and helps to explain critical behaviors [15-17]. According to percolation theory, the reliance on key influencers or nodes is not the most efficient or even the best method for widespread dissemination of public health campaigns. Rather than using high-visibility nodes to facilitate outreach, percolation theory teaches that less influential nodes are more likely to facilitate dissemination of important information to reach critical thresholds and to have widespread impact [17]. Percolation theory modeling of networks can determine key drivers that increase dissemination by solving for R°, which represents the tipping point for widespread dissemination of information. Optimizing the impact of influencers requires facilitating connectivity by understanding potential barriers and reducing their impact. Once these barriers are known, modeling can then be used to guide key decision-making and implement the most effective strategies [18]. A combination of SMS text messaging and social media optimization is more likely to be the most effective approach to public health campaigns. Chatbots can then be incorporated to manage responses to invitations when they are received [19].

A previous study [20] identified the following variables to be key to enhancing response rates in an SMS text messaging campaign:

1. Time of year and influence of seasonal travel, national holidays, and religious, cultural, and social gatherings;
2. Lag between SMS text message receipt and the requested time to respond should be as short as possible to reduce interest decay;
3. Family unit differences between citizens (eg, Kuwaitis) and noncitizens and their potential nodes;
4. Predictive reliability of the screening tool used for enrollment;
5. Monitoring of the survey to identify nodes with positive feedback when identified;
6. Identifying node-to-node engagement and facilitating engagement;
7. Geographic locations of survey recipients and nodes;
8. Demographics of citizens and noncitizens and country of origin;
9. Reliability and program compliance of identified study candidates;
10. Ensuring availability of the needed resources for verification inquiries;
11. Timely reply to submitted surveys through artificial intelligence and chatbots;
12. Feedback on the program with suggestions for improvement for both recruitment efforts and active program opinions;
13. Dissemination of milestones and benefits of the program;
14. Tracking of survey acceptances (“yes,” “no,” and no response); and
15. Cost of the program and economic yield modeling.

This pilot study was conducted to develop a model outreach program that leverages the widely available smartphone for population-based screening, automated program enrollment, and a customized program app with motivational messaging. The goal of the study was to reinforce healthy behavior choices. Resources from the Dasman Diabetes Institute (DDI), a well-known resource for diabetes care in Kuwait, were used to track variables to allow for network analysis. This study was intended to refine strategies and to identify network metrics that could be used to enhance the impact of social media in a diabetes-related health campaign.

## Methods

### Ethical Considerations

Ethics approval for the study was obtained from the DDI’s local Research Ethical Committee (study number: RAHM 2017001) and the Harvard Medical School Institutional Review Board (protocol mumber: 2017P000275/PHS). The study data were deidentified for the US-based investigators and therefore did not require any further review for the secondary analysis. All participants consented to participation in this study prior to enrollment. The original approval by the DDI Research Ethical Committee included using the study data for secondary analysis without additional consent. The investigators based at the DDI kept all phone numbers and data behind a secure firewall compliant with Kuwaiti personal health information regulations as verified by the institution’s institutional review board. No compensation of any kind was provided to the participants enrolled in this study.

A custom-developed novel smartphone app was used to administer a prediabetes scored screening test aimed at identifying individuals at high risk for developing T2DM using SMS text messaging. The CDC’s prediabetes screening questionnaire was translated into Arabic. The program polled cell phone numbers using a database provided by the national cell phone company in Kuwait. Data were collected on secure servers designed to comply with institutional protected health informational security requirements. Participants found to be at elevated risk were encouraged to have further confirmatory diagnostic blood tests at the DDI, which included measuring fasting plasma glucose level, a hemoglobin A_1c_ test, and an oral glucose tolerance test.

### Study Design

We undertook a pilot study to study the impact of SMS text messaging to the users of smartphones in the 6 main governorates in Kuwait. The pilot study collected data to develop a model that could be used for much larger studies aimed to help identify and assist persons at risk of developing T2DM. The data were used to identify key variables that can be used to optimize response rates using the percolation theory equation. The screening program also offered instructions for obtaining confirmatory diagnostic blood testing once it was determined that an individual was at high risk for T2DM.

### Study Participants

This study attempted to recruit adults aged 21 years and older, residing in Kuwait, who owned a compatible smartphone (either iPhone or Android). Five SMS text messaging campaigns were implemented between October 2017 and December 2018. SMS text messages were sent in Arabic and English inviting participation in a study if the recipients were interested to know their own risk of developing T2DM. The following text message was sent: “Are you interested in knowing the risks of developing diabetes? Your data will be used for research purposes. If interested, reply YES to this message.” Candidates were identified as a random subsample provided by the national mobile phone company. The company sent the SMS text messages randomly to 36,000 phone numbers distributed evenly and uniquely without duplication in the 6 main governorates in Kuwait for each of the 5 campaigns; 180,000 SMS text messages were sent in total. Those who were interested replied in the affirmative to the SMS text message. If the participant agreed to participate in the study, a link to an online CDC-validated prediabetes screening test, which measures the risk of developing T2DM, was transmitted to their mobile devices. The questionnaire comprised a simple self-assessment that included the following questions in both English and Arabic:

1. How tall are you? How much do you weigh? (This will establish your Body Mass Index.)
2. Is your Body Mass Index (BMI) greater than 27? Yes or no.
3. How old are you?
4. Do you have a mother, father, sister, or brother with diabetes? Yes or no.
5. Are you physically active? (physically active 20 minutes a day, 3 times per week). Yes or no.
6. Are you male or female? If female have you ever given birth to a baby that weighed more than 9 pounds (or 4 kilogram) and have you ever had diabetes while pregnant?

The test was scored by the app, with possible scores ranging from 0 (no risk) to a maximum of 22 (extreme risk) [20]. The score was based on risk factors of weight (people with higher BMIs have a higher risk of T2DM), older age, family history of diabetes, inactivity, and gender (more men than women have undiagnosed diabetes). Each response was weighted, and the score was summarized. Those scoring ≥9 were classified as high risk for developing T2DM.

Participants who decided to forward the messaging could verify the origin of the SMS text message with the study team located at the DDI through a provided phone number. After verification, the respondent could forward the survey through social media to an acquaintance who might be interested. Completed surveys were received and accessed by research investigators through a research electronic data capture platform called REDCap, which is a browser-based database. Eligible and willing respondents who scored ≥9 points on the CDC questionnaire were contacted and asked to participate in a future diabetes preventive intervention program. All willing respondents had lab work done to rule out the presence of diabetes. Those qualified for enrollment with risk of developing T2DM were sent for laboratory confirmation. If the respondent’s laboratory results confirmed the presence of diabetes but the participant did not meet the inclusion criteria or did not want to participate, referrals were made for treatment instead.

Data were collected on each campaign, including the time of year, length of time that each campaign lasted, occurrence of holidays during the campaign, and seasonal variations. The impact of social media and forwarding to friends and family was determined through examination of the responses to the survey from phone numbers that were not included in the original database of SMS text message invitations. The social media multiplying effect was calculated by using the number of known phone numbers receiving the SMS text message and the number of replies from unknown recipients. In addition, the specificity of the CDC screening tool was also evaluated.

## Results

Because of the seasonal climate changes, Kuwaitis typically leave the country at the beginning in May and return in September or even later when the climate becomes more hospitable. Temperatures and key religious observances associated with the campaign dates are noted in Table 1. Throughout the 5 campaigns, a total of 180,000 SMS text messages were sent, which targeted about 6% of the adult population in Kuwait (Table 1). In total, 260 recipients of the SMS text message replied “Yes,” of which 153 (58.8%) took the survey. Of the 153 survey participants, 92 (60.1%) were classified as high risk for developing T2DM (ie, scored ≥9; Table 2).

After screening and validation, the demographic data of the respondents who agreed to participate were compiled (Table 3).

**Table 1:** Results of the SMS text messaging campaign.

Campaign number	Time period	Average daily high/low temperature (°C)	Responses received (N=627), n	Surveys completed (N=153), n	Qualified for enrollment^a^, n	Associated dates of Islamic holidays
1	October 2017	31/22	37	13	6	September 21, 2017 (Start of Muharram^b^)
2	February 2018	24	43	22	12	None
3	April and May 2018	April: 32/30; May: 44/33	30	15	7	May 16, 2018 (sStart of Ramadan^c^), June 15, 2018 (Eid al-Fitr^d^)
4	September 2018	37/25	113	76	52	September 11, 2018 (sStart of Muharram^b^)
5	December 2018	16/14	37	27	15	None
Social media	N/A^e^	N/A	367	N/A	N/A	N/A

^a^Participants qualified for enrollment if they were at risk of developing type 2 diabetes but did not yet have laboratory confirmation of diabetes.

^b^Muharram is Islamic New Year.

^c^Muslims practice 1 month of fasting during Ramadan.

^d^Eid al-Fitr is the feast marking the end of Ramadan.

^e^N/A: not applicable.

**Table 2:** Regional and campaign characteristics of participants.

Campaign number			Gender, n				Nationality, n				Newly diagnosed with diabetes, n				Attended orientation, n		Installed app, n
			Male		Female		GCC^a^		NGCC^b^		Male		Female				
**1**											1		0		0		—^c^
	Qualified	0		0		0		0									
	Not qualified	1		0		0		1									
**2**											0		2		1		—
	Qualified	1		0		3		0									
	Not qualified	0		2		0		0									
**3**											0		1		2		2
	Qualified	2		0		1		2									
	Not qualified	0		1		0		0									
**4**											0		2		18		18
	Qualified	10		17		13		15									
	Not qualified	0		2		0		1									
**5**											0		1		0		—
	Qualified	5		3		4		3									
	Not qualified	0		1		0		1									
**Friends and family**											4		4		6		6
	Qualified	16		52		50		26									
	Not qualified	4		4		0		0									

^a^GCC: countries belonging to the Gulf Cooperation Council, which include Saudi Arabia, Bahrain, Kuwait, Qatar, Oman, and the United Arab Emirates.

^b^NGCC: countries not belonging to the Gulf Cooperation Council.

^c^Not available.

**Table 3:** Demographic information of the participants.

Variable		Participants (N=68)
**Age range (years), n (%)**		
	21-45	39 (57)
	45-65	25 (37)
	>65	4 (6)
**Gender, n (%)**		
	Female	37 (54)
	Male	31 (46)
**BMI, mean (SD)**		29.6 (5.0)
	<25, n (%)	10 (15)
	≥25 and <30, n (%)	33 (48)
	≥30 and <40, n (%)	23 (34)
	≥40, n (%)	2 (3)

Unexpectedly, 367 (70.6%) surveys were completed by individuals who were not sent the original SMS text message. These individuals completed the survey after the SMS text message was forwarded to them by family members, friends, or other acquaintances, and of these, 309 (84.2%) were found to have a risk score of ≥9. Of the total respondents, 229 (44%) agreed to be contacted and 121 (23.3%) consented and enrolled in a diabetes prevention study. Surprisingly, long past the time that the SMS text messages were sent, completed surveys continued to appear. Anecdotal information suggests that a subset of contacted respondents circulated the SMS text message survey to family members and friends primarily through other social media platforms (predominantly WhatsApp) after establishing the legitimacy of the survey by contacting the sponsoring institution.

The forwarded messages substantially increased the number of candidates enrolled in the treatment phase or second step in this study. The responders to forwarded SMS text messages were more likely to be at risk for T2DM, suggesting that the recipients forwarded messages to acquaintances who were thought to be at risk of developing T2DM.

## Discussion

### Implications of the Study Results

Our previous study using SMS text messaging in Kuwait resulted in a low response rate for these campaigns [21]. We tracked variations in response rates and identified correlations with holidays, seasons, program administrative support, and the accuracy of our screening tools. Along with seasonal and religious holidays, we noted a surprising multiplying effect when SMS text messaging was combined with messaging on social media with friends and family members.

As part of this pilot study, we collected data that could be used as parameters in developing a strategy to maximize influence through percolation theory by quantifying influencers or nodes. Our approach used SMS text messaging to identify people at risk and used marketing tools, such as the DDI website, to improve response rates. The results of this study suggest that SMS text messaging has the potential to become a useful, effective, and inexpensive method of reaching a subpopulation of individuals at risk for T2DM and lays the groundwork for identifying influencers of social networks that can be used to facilitate and significantly improve response rates in public health campaigns.

The true potential of our findings will require the development of features that can provide data for the metrics investigated in this study that would allow for continued monitoring and the running of percolation theory predictive scenarios. The program will also need to facilitate social media contacts and encourage friends and family forward messaging by developing the needed features to facilitate aestheticization of the SMS text messaging program messages. Past studies have demonstrated that SMS text messaging and social media can be used as effective health care reminders. Motivating messages designed to encourage behavioral change, including health promotion and disease prevention, can also be effective. Unfortunately, these approaches suffer from relatively low response rates. This study illustrates the potential of reaching a large population of smartphone users in the Middle East to screen for health care risks who might not have otherwise been identified or informed through existing databases of cell phone numbers. Further efforts are needed to not only refine the initial messaging methodology but also to facilitate the incorporation of social media into this public outreach program to take advantage of the multiplying potential and promoting the long-lasting residual impact of any intervention.

Our data suggest that SMS text messaging campaigns could serve a secondary purpose as a social search tool to identify interested facilitators. These facilitators can then be asked to forward the message through social media to their contacts. Factors involved in facilitating social media growth will include contact verification and mentoring resources at the sponsoring institution, imbedded contact tracing ability, and a formal recognition of champions that have become important allies.

Carefully scripting the message, optimizing the supporting infrastructure to facilitate social media, and incorporating chatbots while planning for points identified in this paper will likely improve response rates in subsequent studies and provide more cost-effective public health initiatives. Data for each of these metrics will be used to calculate the probability of a response by using the Bayes theorem, modifying it for dependent variables that will be defined in real time and incorporated into subsequent studies:



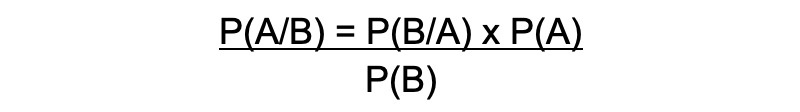



where P is the probability of response, and A is the response given the occurrence of variable B (eg, variables 1-13 noted in the introduction).

A previous study in Uganda used SMS text messaging as a tool for health education [22]. Their approach was to send 13 questions related to HIV/AIDS to 10,000 mobile users over a narrow time window of 1 month. The response rate was 2%. Despite the low response rate, they acknowledged the importance of the use of mHealth to reach numerous mobile users as an integrated tool of a health campaign. Their reported findings are similar to the results in this study. Because the use of SMS text messaging campaigns for health purposes is relatively new, future efforts are needed to help improve public acceptability by using appropriate marketing strategies and defining and optimizing important variables that support improved response rates.

The annual cost of T2DM worldwide is highly variable. Yearly costs including both direct and indirect expenses range widely from the lowest economic impact in low-income countries to costs in the western world that exceed US $16,000 per year [23]. In order to sustain public health initiatives, countries that have limited health care resources need to show a measurable return on investment. A major finding of this study is that SMS text messaging can not only recruit candidates for intervention but can reach others by being forwarded by family members and friends through available social media resources. This appears to be a cost-free means to improve contact with those who may be at risk for developing a chronic disease.

Although smartphones are a widely available access point for public health campaigns, potential concerns should be noted. These include the potential breach of protected health information, mistrust in the source of messaging, and penetration of various firewalls that protect the infrastructure needed to support these types of studies. In the age of misinformation on the internet, obtaining reliable health information is important. Future trials should consider ways to assist the receiver of any SMS text messaging information to know when and how any information that is being sent can be verified and trusted. This is an important consideration among those seeking help in the management of diabetes.

### Strengths and Limitations

To our knowledge this is the first study to use SMS text messaging to reach a wide audience of mobile phone users through the primary campaign and secondary forwarding of SMS messages in the Middle East to facilitate the identification of risk of diabetes and candidates for follow-up care.

There are several limitations of this study that need to be discussed. First, this was not a controlled trial, and we were unable to directly track the benefit of social media and SMS text messaging on the management of diabetes. Second, using the CDC questionnaire, which was designed for use in the United States, in Kuwait likely missed some at-risk patients. Although finding undiagnosed people with diabetes is certainly useful, the cutoff score of 9 likely missed some eligible candidates. As part of this study, we included an Arabic version of the questionnaire that offered a greater opportunity to identify at-risk candidates. Further validation is needed to determine the benefit of the cutoff score and the benefit of an Arabic translation for this specific population. Third, this study did not assess education level or digital competencies. This would be important for future studies, as there are likely many individuals at risk for T2DM who have difficulty with the use of digital health or computerized information technology due to limited education or a lack of training in computer technology.

Despite these limitations, this study demonstrates that future use of SMS text messaging health campaigns could support the incorporation of social media for mass screening. The findings of this study suggest that use of SMS text messaging in conjunction with other popular social media platforms can offer a feasible, accessible, and cost-effective means of reaching individuals at risk of developing T2DM while enriching the pool of respondents for the targeted intervention. Historically, compliance with treatment recommendations has been low. This communication approach has the potential to become a strategy to reinforce the therapeutic relationship between a patient and their providers. Future studies will be encouraged to identify those factors that would increase the response rate to survey invitations and identify ways for users to benefit from technologies, including chatbot responses.

## Abbreviations

CDC: Centers for Disease Control and Prevention
DDI: Dasman Diabetes Institute
GCC: Gulf Cooperation Council
mHealth: mobile health
T2DM: type 2 diabetes mellitus

## Data Availability

All data generated and analyzed are included in this article and a previously published article [21]. Raw data sets analyzed during this study are available from the corresponding author on reasonable request.
